# The Gut–Brain–Microbiota Connection and Its Role in Autism Spectrum Disorders

**DOI:** 10.3390/nu17071135

**Published:** 2025-03-25

**Authors:** Ewelina Młynarska, Ewelina Barszcz, Emilian Budny, Agata Gajewska, Kacper Kopeć, Jakub Wasiak, Jacek Rysz, Beata Franczyk

**Affiliations:** 1 Department of Nephrocardiology, Medical University of Lodz, ul. Zeromskiego 113, 90-549 Lodz, Poland; 2 Department of Nephrology, Hypertension and Family Medicine, Medical University of Lodz, ul. Zeromskiego 113, 90-549 Lodz, Poland

**Keywords:** autism, microbiota, probiotics, gut–microbiota–brain axis, diet

## Abstract

Autism spectrum disorder (ASD) is a group of complex neurodevelopmental conditions with a heterogeneous and multifactorial etiology that is not yet fully understood. Among the various factors that may contribute to ASD development, alterations in the gut microbiota have been increasingly recognized. Microorganisms in the gastrointestinal tract play a crucial role in the gut–brain axis (GBA), affecting nervous system development and behavior. Dysbiosis, or an imbalance in the microbiota, has been linked to both behavioral and gastrointestinal (GI) symptoms in individuals with ASD. The microbiota interacts with the central nervous system through mechanisms such as the production of short-chain fatty acids (SCFAs), the regulation of neurotransmitters, and immune system modulation. Alterations in its composition, including reduced diversity or an overabundance of specific bacterial taxa, have been associated with the severity of ASD symptoms. Dietary modifications, such as gluten-free or antioxidant-rich diets, have shown potential for improving gut health and alleviating behavioral symptoms. Probiotics, with their anti-inflammatory properties, may support neural health and reduce neuroinflammation. Fecal microbiota transplantation (FMT) is being considered, particularly for individuals with persistent GI symptoms. It has shown promising outcomes in enhancing microbial diversity and mitigating GI and behavioral symptoms. However, its limitations should be considered, as discussed in this narrative review. Further research is essential to better understand the long-term effects and safety of these therapies. Emphasizing the importance of patient stratification and phenotype characterization is crucial for developing personalized treatment strategies that account for individual microbiota profiles, genetic predispositions, and coexisting conditions. This approach could lead to more effective interventions for individuals with ASD. Recent findings suggest that gut microbiota may play a key role in innovative therapeutic approaches to ASD management.

## 1. Introduction

Autism spectrum disorder (ASD) represents a heterogeneous group of neurodevelopmental disorders that typically emerge early in life, characterized by difficulties in social communication and distinctive patterns of restricted, repetitive behaviors and interests [1]. ASD is a significant public health concern and the fastest-growing developmental disorder, generating annual costs of USD 236–262 billion in the USA, with an estimated lifetime social cost of approximately USD 3.6 million per individual; by 2029, total social costs are projected to exceed USD 11.5 trillion [2,3]. Severe forms of ASD are linked to a substantial economic burden, parental stress, and numerous co-occurring medical and behavioral issues [2]. ASD affects around 1% of the global population and is diagnosed about four times more frequently in males than females. Additionally, one-third of individuals with ASD have co-occurring intellectual disabilities. The observed increase in prevalence may reflect heightened societal awareness, improved case identification, and evolving diagnostic criteria [4].

The etiology of ASD is multifactorial, involving complex interactions between genetic, epigenetic, and environmental factors, including immune dysregulation [5]. Recent evidence suggests that alterations in the gut microbiota, immune system, and neurodevelopment are closely interconnected and may interact with each other [6]. Studies have reported elevated levels of pro-inflammatory cytokines, including tumor necrosis factor-alpha (TNF-α), interleukin-8 (IL-8), and interleukin-6 (IL-6), as well as increased concentrations of certain anti-inflammatory cytokines, such as interleukin-4 (IL-4) and interleukin-10 (IL-10), in children with ASD [7]. To date, over 100 genes and genomic regions have been implicated in ASD, with a broader range (350–400 genes) potentially contributing to increased susceptibility [8]. Autism exhibits significant phenotypic heterogeneity, complicating the understanding of its genetic basis. While common genetic variants are associated with core autism traits, de novo variants do not show the same correlation. A higher autism polygenic score (PGS) is linked to a lower likelihood of co-occurring developmental disabilities. Among autistic individuals without intellectual disability (ID), PGS is more frequently inherited by females, and SNP heritability is higher in males and those without ID. The analysis of four copy number variant (CNV) groups revealed substantial phenotypic variability within groups and minor differences between them. Additionally, nearly half of the individuals with CNVs who did not meet the full diagnostic criteria for ASD exhibited elevated autistic traits, suggesting a continuum between the autism spectrum and the general population [9,10].

Environmental risk factors, although still under investigation, include advanced parental age, birth complications associated with trauma, ischemia, and hypoxia, as well as, to a lesser extent, maternal obesity, maternal diabetes, and cesarean delivery [11].

ASD is associated with a significant burden of comorbidities, with 74% of individuals experiencing at least one co-occurring condition and more comorbidities on average than their non-ASD siblings. These comorbidities include attention deficit hyperactivity disorder (ADHD), anxiety disorders, epilepsy, obsessive–compulsive disorder (OCD), sleep disturbances, sensory processing issues, motor coordination difficulties, and gastrointestinal (GI) disorders [12]. Among these, gastrointestinal (GI) disorders are particularly prevalent in children with ASD, manifesting as constipation, diarrhea, abdominal pain, and gastroesophageal reflux disease (GERD). GI conditions occur up to four times more frequently in children with ASD than in neurotypical peers, with prevalence rates ranging from 9% to 91% [13]. The severity of GI disorders in children with ASD is closely linked to the intensity of neuropsychiatric symptoms, with more severe GI issues observed in individuals with more pronounced neuropsychiatric symptoms, and vice versa [14].

The gut microbiota, a diverse community of microorganisms inhabiting the gastrointestinal tract, begins to develop in the prenatal period, and disruptions in its composition during the first 2–3 years of life can negatively impact development and increase the risk of future diseases [15]. It plays essential metabolic functions by aiding in the digestion of dietary fibers and producing short-chain fatty acids (SCFAs), which provide energy and exhibit anti-inflammatory properties. It also has protective roles, strengthening the gut barrier through the secretion of mucus, antimicrobial peptides, and immunoglobulin A (IgA). Structurally, it maintains the integrity of the intestinal epithelium and supports cell regeneration [16]. Dysbiosis, characterized by alterations in the gut microbiota composition, has been implicated in the gastrointestinal and neuropsychiatric symptoms of ASD, potentially through mechanisms involving immune dysregulation, increased intestinal permeability, and disrupted gut–brain communication [8]. The gut microbiota composition in children with autism is characterized by a reduced *Bacteroidetes/Firmicutes* ratio, an increased abundance of Lactobacillus species, and a slight elevation in *Desulfovibrio*, which correlates with the severity of autism symptoms [17].

Alterations in the gut microbiota of individuals with ASD, including imbalances in the abundance of Gram-negative bacteria involved in sulfur metabolism, such as an increased presence of *Bilophila* and a decreased abundance of *Desulfovibrio*, may affect the availability of sulfur metabolites and disrupt the biochemical homeostasis of the body [17,18]. These dysfunctions may significantly impact sulfur amino acid (SAA) metabolism, which is implicated in ASD pathogenesis. Individuals with ASD exhibit lower levels of methionine, cysteine, and S-adenosylmethionine (SAM) in body fluids, alongside elevated S-adenosylhomocysteine (SAH) compared to neurotypical individuals. These metabolic disturbances may impair remethylation and methylation capacity, potentially affecting nervous system function and exacerbating ASD symptoms [19].

In this narrative review, the literature was selected based on searches conducted in databases such as PubMed and Google Scholar. Both original research articles and systematic reviews relevant to the topic were included, with the selection criteria based on their recency and significance to the subject of study. Additionally, the articles cited in the tables comprised both original studies and review articles, including research conducted on both humans and animals.

## 2. Gut Microbiota and Gut–Brain Axis

A growing area of research in recent years has focused on the gut microbiota, the gut–brain axis (GBA), and their influence on bodily functions, including brain activity and cognitive function. The human gut microbiota and its host share a mutually beneficial relationship: the host provides a suitable environment and nutrients, while the microbiota supports health by aiding nutrient absorption. This harmonious relationship, known as eubiosis, is essential for overall well-being and stands in contrast to dysbiosis—a state of imbalance in the microbiota’s composition and function [20,21].

**Figure 1:**
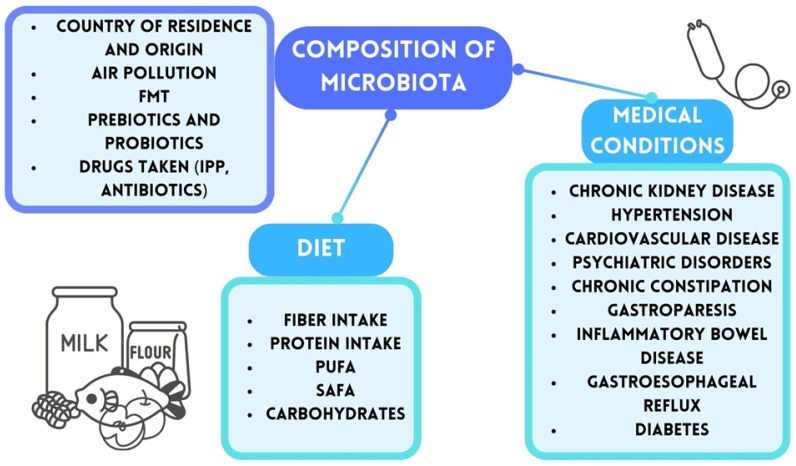
Factors affecting microbiota composition. IPP—protein pump inhibitors; FMT—fecal microbiota transplant; PUFA—polyunsaturated fatty acids; SAFA—saturated fatty acids.

The human gut microbiome is estimated to contain over 100 trillion bacteria, with their density increasing along the digestive tract and reaching approximately 10^12^–10^14^ bacteria per gram of tissue in the colon [20,22]. The collective genetic material of these microbes is 100 times greater than the human genome, and their combined mass is estimated at 1 to 3 kg [23,24]. Microbiota development begins in the womb and is influenced by factors such as mode of delivery—vaginal or cesarean—and the infant’s diet, particularly breastfeeding versus formula feeding [25]. While short-term dietary changes account for about 20% of microbiota variability, long-term eating habits exert a more profound impact [26,27]. Diet also determines the predominance of specific bacterial species, leading to two primary enterotypes: one dominated by *Bacteroides*, prevalent in industrialized regions with low-fiber, high-fat diets, and another dominated by *Prevotella*, more common in regions with high-fiber, minimally processed diets [28,29]. Additionally, some researchers have identified a third enterotype, which is characterized by the predominance of *Ruminococcus* [30]. It is important to note that the makeup of the microbiota is unique and influenced by various circumstances, including country of residency and environmental contamination. Any illnesses or medical disorders present are also critical. An overview of the variables impacting the microbiome makeup in adults is provided in Figure 1 [31,32,33,34,35,36,37,38,39].

### 2.1. Gut–Brain Axis and Bacterial Metabolites

GBA facilitates bidirectional communication between the gut microbiota and the brain, making eubiosis essential for human health. This system encompasses interactions between the gut-associated immune system, the enteric nervous system (ENS), the vagus nerve, and microbial-derived products, including neurotransmitters, SCFAs, and bile acid derivatives [40,41]. The autonomic nervous system and ENS regulate gut secretion, motility, and immune responses, all of which influence microbiota composition [42].

The endocrine pathway serves as a critical link between ASD and the gut–brain axis, primarily through hormone secretion by enteroendocrine cells (EECs). These cells produce various hormones, including serotonin, glucagon-like peptide-1 (GLP-1), and peptide YY (PYY). GLP-1 and PYY communicate with the brain via the vagus nerve, affecting appetite and eating behaviors. Disruptions in endocrine signaling due to an imbalanced gut microbiome may contribute to ASD-related behaviors and altered feeding patterns [43].

The integrity of GBA relies on the intestinal barrier, which maintains separation between the intestinal lumen and the rest of the body, regulating solute and fluid exchange. Dysbiosis can compromise this barrier, leading to increased intestinal permeability, commonly referred to as “leaky gut” syndrome (LGS). LGS allows microbial components like lipopolysaccharide (LPS), toxic metabolites, and inflammatory agents to enter the bloodstream [44,45]. Within the body, LPS is recognized by Toll-like receptors (TLRs), particularly TLR4, which belong to the pattern recognition receptors (PPR) family. These bacterial toxins activate nuclear factor κB (NF- κB), which, in conjunction with TLR4, regulates cytokine expression. This process results in the release of pro-inflammatory molecules and interleukins, such as TNF-α, IL-6, IL-8, and IL-12, contributing to both local and systemic inflammation [46,47].

SCFAs, including butyrate and propionate, produced by gut bacteria via fiber fermentation, exert widespread effects. They modulate the immune system activity, regulate appetite, enhance calcium absorption, and contribute to glucose homeostasis [48,49]. Studies indicate that autistic children exhibit lower overall SCFA levels, with a particularly pronounced reduction in butyrate [50]. SCFAs help preserve blood–brain barrier (BBB) integrity, preventing neurotoxic substances from entering the brain [51]. Furthermore, SCFAs cross the BBB and influence early brain development by regulating the production of serotonin and dopamine [50,52]. Two free fatty acid receptors, FFA2 and FFA3, located in various brain regions, are bound by SCFAs [53].

Priopionic acid (PA) has been shown to promote neuroregeneration through mechanisms involving free fatty acid receptor signaling and the inhibition of class I/II histone deacetylase (HDAC) activity [54]. Additionally, SCFAs possess neuroprotective properties and, through epigenetic modifications, enhance memory formation and neural plasticity [49,50,55]. In patients with multiple sclerosis (MS), long-term PA supplementation has been linked to positive outcomes, such as a lower annual relapse rate and slower disease progression. Additionally, PA has been shown to modulate the immune system by significantly increasing the number of functional Treg cells while reducing Th1 and Th17 cell populations [56].

Butyrate plays a crucial role in regulating anti-inflammatory responses by controlling the expression of forkhead box protein P3 (Foxp3), essential for suppressing excessive inflammation [57].

Other SCFAs, such as acetate, have been implicated in modulating ASD-like behaviors. Studies indicate that oral acetate supplementation can reverse social deficits and alter gene expression in the medial prefrontal cortex in mice carrying a deletion in exons 4–22 of the Shank3 gene [58]. Moreover, acetate is essential for lipid metabolism and glucose regulation [59,60]. In mice, dietary fructose is metabolized by gut microbes into acetate, serving as a source of acetyl-CoA for lipid synthesis [61]. Acetate also exhibits anti-inflammatory properties, suppressing NF-κB activation and lowering the production of pro-inflammatory mediators like lipopolysaccharide-induced TNF-α [62].

Another gut-derived metabolite, Trimethylamine *N*-oxide (TMAO), is primarily produced by the small intestinal microbiota from dietary precursors, including choline, betaine, carnitine, and deoxycarnitine, which are abundant in meat, eggs, and dairy products. TMAO has been implicated in cardiovascular diseases, contributing to platelet hyperreactivity, increased thrombosis risk, and endothelial dysfunction [63,64,65]. Additionally, TMAO promotes the activation of the NLRP3 microglial inflammasome, exacerbating neurological damage in ischemic stroke [66]. However, the effects of TMAO on brain function remain inconclusive, as a large-scale study found no significant association between TMAO levels and cognitive decline [67]. Similarly, in the context of ASD, research findings are inconsistent [68,69].

Nonetheless, indole-3-propionic acid (IPA), one of the primary tryptophan metabolites produced by the gut microbiota, has been suggested to influence ASD susceptibility in offspring. Notably, supplementing microbiota-derived IPA has been proposed as a potential intervention for fetal-origin ASD [70]. The gut microbiota and its metabolites play a critical role in tryptophan metabolism. The majority of tryptophan is metabolized through the kynurenine pathway, a process modulated by bacterial LPS and pro-inflammatory cytokines, which stimulate indoleamine 2,3-dioxygenase and tryptophan 2,3-dioxygenase, the key enzymes involved in this pathway. Consequently, serotonin synthesis from tryptophan is reduced. Under inflammatory conditions, tryptophan metabolism shifts towards increased kynurenine levels, leading to an imbalance in its downstream metabolites (TRYCATs), such as quinolinic acid (QUIN) and kynurenic acid (KYNA). This disruption can cause structural and functional damage to the central nervous system, as the QUIN overactivation of NMDA receptors leads to excessive stimulation, while excessive KYNA, despite its neuroprotective role, contributes to neurodegeneration and cognitive decline [71,72,73].

Beyond SCFAs and other metabolites, the gut microbiota influences central nervous system (CNS) activity by modulating the production and metabolism of neurotransmitters and bioactive molecules, including acetylcholine, catecholamines, histamine, and adenosine [74]. However, low-grade systemic inflammation associated with LGS damages the BBB, a critical structure responsible for brain homeostasis and proper neuronal function [75]. Increased BBB permeability has been associated with heightened microglial activity and a reduction in astrocyte numbers and functionality [52,76]. These changes align with post-mortem studies of autistic individuals, in which studies have observed increased microglial activation and structural abnormalities [77].

Astrocytes, which express TLR4 receptors, can initiate a pro-inflammatory response upon activation by LPS [78]. Additionally, astrocytes regulate levels of gamma-aminobutyric acid (GABA) and glutamate by facilitating their reabsorption. Brain inflammation caused by these disruptions can lead to abnormal synapse function and stimulate the release of vasopressin, a biomarker of ASD known to affect social behavior [79].

Another crucial aspect of bacterial influence on neuroinflammation involves outer membrane vesicles (OMVs)—small, spherical structures released by Gram-negative bacteria. OMVs transport various cargo molecules, including the following: LPS, outer membrane proteins (OMPs), lipooligosaccharides (LOS), phospholipids, peptidoglycan (PGN), periplasmic components, and virulence factors, such as enzymes and toxins [80]. It was shown that OMVs from *Porphyromonas gingivalis* or *Treponema denticola* stimulate pro-inflammatory responses by increasing TNF-α and IL-8 production and triggering IL-1β secretion. This leads to monocyte and macrophage activation, causing inflammatory cell death and tissue damage both in vitro and in vivo [81]. Another study reveals that *H. pylori* OMVs can bypass BBB and reach the brain, where they are absorbed by astrocytes. This interaction activates glial cells, disrupts neuronal function, and accelerates amyloid-β pathology, ultimately contributing to cognitive decline [82].

PGN, a bacterial cell wall component, can cross the BBB and is recognized by PGN-sensing molecules (PGLYRP2 and NOD1), which are highly expressed in neurons of the prefrontal cortex, hippocampus, and cerebellum. These findings suggest that PGN may directly impact neuronal function [83,84].

Chronic systemic inflammation linked to LGS also disrupts the hypothalamus–pituitary–adrenal (HPA) axis, a key system for providing high-energy fuels such as glucose, amino acids, and free fatty acids to support immune activity. This disruption results in the increased production of glucocorticoids and catecholamines, causing hypercortisolemia and HPA axis overactivation, often accompanied by impaired glucocorticoid receptor function [85,86]. Studies on germ-free mice have demonstrated that the absence of gut microbiota leads to an exaggerated HPA stress response. Chronic activation of this pathway can contribute to increased anxiety, impaired cognitive function, and heightened stress reactivity in individuals with ASD [87]. Furthermore, increased intestinal permeability can activate T-cells, potentially triggering autoimmune disorders in the gut or other organs as these activated lymphocytes circulate throughout the body [88].

As previously mentioned, dysbiosis, LGS, and GBA dysfunction are common in numerous diseases. Although the underlying mechanisms remain complex and are still under investigation, they are frequently linked to bacterial translocation, chronic low-grade inflammation, and substances derived from the microbiota.

### 2.2. Neurotransmitters

Neurotransmitters play a crucial role in CNS function and neurodevelopment. Some of the most significant neurotransmitters include catecholamines (adrenaline, noradrenaline, and dopamine), serotonin, GABA, adenosine triphosphate (ATP), and histamine. Recent findings suggest that catecholamines also participate in gut immune regulation, albeit in an unexpected manner. These compounds appear to suppress the intestinal mucosal immune response while simultaneously enhancing the virulence and aggressiveness of certain bacterial species [43]. In children with autism, increased levels of various soluble factors have been identified in the bloodstream. Neurotransmitters synthesized in the gut interact with epithelial cells in the intestinal lining, triggering the production of bioactive molecules that can influence the CNS. This process occurs either through the release of these molecules into the circulatory system or by stimulating afferent neurons. Among children with autism, elevated levels of serotonin—a crucial neurotransmitter for both the brain and the gut—have been detected. In the gastrointestinal system, serotonin regulates processes such as motility, pain sensitivity, and secretion, while in the brain, it plays an essential role in mood regulation and cognitive functions.

Microorganisms such as *Candida*, *Streptococcus*, *Escherichia*, and *Enterococcus* are known contributors to serotonin production [52]. These species within the gut microbiota contain enzymes that play a key role in regulating tryptophan metabolism, leading to the production of compounds such as serotonin, kynurenine, and indole derivatives. By modulating tryptophan availability, the gut microbiota can influence serotonin levels in the brain.

Acetylcholine, produced by *Lactobacillus* spp., and dopamine, synthesized by *Bacillus* spp. and *Serratia* spp., are other neurotransmitters that may be elevated and implicated in ASD [89]. Additionally, increased concentrations of GABA, an inhibitory neurotransmitter, have also been observed in children with autism. Microbial species including *Bacillus*, *Enterococcus*, *Escherichia*, *Saccharomyces*, and *Streptococcus* are recognized as producers of GABA [52]. Research indicates that autistic brains exhibit an imbalance between excitatory and inhibitory (E-I) signals. It is hypothesized that disturbances in GABAergic neuron activity play a role in the development of the disorder [90].

Patterns of hyper- and hypo-glutamine observed at different developmental stages support the hypothesis that neurotransmitter imbalances play a role in the pathogenesis of ASD. Research on *N*-methyl-D-aspartate receptor (NMDAR) and alpha-amino-3-hydroxy-5-methyl-4-isoxazolepropionic acid receptor (AMPAR) antagonists has shown potential clinical benefits for individuals with ASD. Additionally, the excitatory glutamate pathway appears to contribute to the etiopathogenesis of ASD by functioning as part of the gut–brain axis and facilitating trans-synaptic signaling [89].

Children with ASD are also reported to have significantly decreased levels of metabolites such as glutathione, homocysteine, methionine, and S-adenosylmethionine in their blood. S-adenosylmethionine serves as a crucial methyl donor in the body, with homocysteine acting as its precursor, indicating potential disruptions in sulfur metabolism. Glutathione plays a key role in alleviating oxidative stress within cells. These metabolic alterations increase the vulnerability of autistic children’s cells to oxidative stress [52].

## 3. The Association Between Gut Microbiota and the Development and Progression of Autism Spectrum Disorders (ASD)

### 3.1. The Basic Elements of Gut Microbiota and the Characteristics of Their Metabolic Products

The impact of the gut microbiome on human health has been extensively studied by researchers over the years [89]. Studies suggest a potential imbalance in the intestinal microbiota composition of individuals with ASD, with significant differences in the relative abundance of specific bacterial taxa compared to healthy controls. The most notable alterations included the different prevalences of *Bacteroidetes/Firmicutes*, *Bifidobacterium*, *Faecalibacterium*, *Clostridium*, *Enterobacteriaceae*, *Verrucomicrobia*, *Fusobacteria*, *Escherichia coli*, *Lactobacillus*, *Streptococcus*, *Enterococcus*, *Prevotella,* and *Akkermansia*. These findings provide a comprehensive understanding of the dysbiotic characteristics of the intestinal microbiota in individuals with ASD, offering novel insights into potential diagnostic and therapeutic interventions for this condition [89,91,92].

Extensive research has demonstrated that gut microbiota can produce a wide range of metabolic products, some of which influence the human nervous system via the gut–brain axis. Examples of these metabolic products include SCFAs, which are produced by *Lactobacillus*, *Bifidobacterium,* and *Prevotella* [93]. Furthermore, certain bacterial strains, including Lactobacillus and Bifidobacterium, have been observed to modulate the production of neurotransmitters such as GABA [94]. It has been hypothesized that elevated levels of S100 calcium-binding protein beta subunit (S100B) may contribute to ASD development by promoting neuroinflammation and interacting synergistically with pro-inflammatory cytokines. Studies have shown that levels of this protein are significantly elevated in individuals with autism. In addition, an in vivo study in mice showed that the biodiversity of the gut microbiota increases with S100B levels. *Firmicutes*, which includes *Lactobacillus*, and *Bacteroidetes*, which includes *Barnesiella* and *Butyricimonas* spp., influence S100B values [95]. A dedicated chapter of this review explores the molecular mechanisms linking gut microbiota to ASD development.

### 3.2. A Comparative Analysis of the Gut Microbiota in Patiens Diagnosed withASD and a Healthy Population

Numerous scientific studies have demonstrated a correlation between elevated levels of certain gut bacteria and the presence of ASD. This correlation is presented in Table 1. Studies has shown a significant increase in the prevalence of *Clostridium* bacteria in ASD patients compared to the general population [8,50,52,96,97,98,99,100,101,102,103,104,105,106,107,108,109]. Additionally, studies suggest a potential increase in the prevalence of *Lactobacillus* in the fecal samples of patients diagnosed with ASD [8,18,50,96,97,98,102,103,106,109,110,111]. In addition, the research by Fattorusso et al. indicated the potential for an increased abundance of *Caloramator* and *Sarcina* in ASD cases [8]. This relationship has been documented in other scientific studies [50,96,97,98]. Moreover, an elevated abundance of *Ruminococcaceae*, *Enterobacteriaceae*, *Pasteurellaceae*, and *Lachnospiraceae* families has been observed in the digestive tract of individuals with ASD [8,50,52,96,97,98,101,105,110,111]. In their study, Srikantha et al. have demonstrated a possible escalation in the counts of genera *Barnesiella*, *Odirobacter*, *Porphyromonas*, *Anaerofilum*, *Parasutterella*, *Aeromonas*, *Pseudomonas*, and *Alcaligenaceae* in patients diagnosed with ASD [52]. A recent study has shown that the prevalence of bacteria from the genus *Dorea* is higher in patients with ASD in comparison to healthy individuals [18,52]. In addition, an overgrowth of *Ralstonia*, *Burkholderia*, and *Peptostreptococcus* has been observed in patients diagnosed with ASD [112]. Furthermore, the presence of *Alistipes* may be observed at elevated levels in patients diagnosed with ASD [50,89,96,97,98]. Furthermore, the findings of independent studies have indicated a potential increase in the abundance of *Collinsella*, *Eggerthella*, *Haemophilus*, *Klebsiella*, and *Phyllobacterium* bacteria in the intestines of individuals diagnosed with ASD compared to the general population [18,102,104,111]. In addition, a scientific study of bacteria in the *Eggerthella* genus revealed an increase in the prevalence of two distinct taxa: *Eggerthella lenta* and *Eggerthella lenta* DSM 2243 [104]. It was also observed that the abundance of the genus *Faecalibacterium*, particularly the *Faecalibacterium prausnitzii* species, should be lower in healthy individuals compared to patients diagnosed with ASD [52,89,101,107,109,113]. Moreover, a decrease in the populations of *Akkermansia* and *Sutterella*, with a particular emphasis on *Akkermansia muciniphila* species, was observed in healthy controls [52,89,107,109,110]. An increasing number of studies indicate that the population of *Desulfovibrio* could exhibit an increase in cases of ASD [50,96,97,98,103,106,107,109].

Research indicates that certain bacterial species within the gastrointestinal tract may be reduced in individuals diagnosed with ASD. This correlation is illustrated in Table 2. Studies indicate that the prevalence of certain bacterial families, including *Coriobacteriaceae*, *Actinomycetaceae*, and *Bifidobacteriaceae*, has been observed to decline in individuals with ASD compared to those in the general population [8,101]. A notable decrease in the prevalence of bacteria from the *Veillonellaceae* and *Streptococcaceae* families was observed in patients with ASD. Additionally, there was a corresponding reduction in the number of bacteria from the *Veillonella* and *Streptococcus* genera, which are classified within these families [8,18,52,101,107,112,114,115]. Moreover, research studies by Kushak et al. and Srikantha et al. indicate a possibly greater presence of *Neisseria*, *Devosia*, *Escherichia*, *Fusobacterium*, *Sporobacter*, and *Subdoligranulum* in individuals without ASD [52,112]. In addition, the presence of *Staphylococcus*, *Coprococcus*, *Lactococcus*, and *Flavonifractor* may be detected in lower quantities [8,52,107,111,114,115]. A multitude of scientific studies have reported a potential decrease in the abundance of *Bifidobacterium* in individuals diagnosed with ASD [52,89,101,102,103,106,107,113]. Moreover, recent scientific research studies have shown a lower prevalence of the bacteria *Blautia*, *Dialister*, and *Bilophila* [18,103]. Notably, Soltysova et al. demonstrated that the rise in *Bilophila* bacteria was more pronounced in adults with ASD, while in children, the number of bacteria of this genus was diminished [116]. Furthermore, a decrease in the abundance of *Eubacterium* and *Enterococcus* populations within the gastrointestinal tracts of patients diagnosed with ASD has been observed when compared to healthy populations [52,107,109].

Studies provide conflicting data regarding the increase or decrease in the population of intestinal bacteria in patients with ASD. The previous information is displayed in Table 3. Coretti et al. described a decrease in the populations of *Corynebacterium* and *Actinomyces* in individuals with ASD; nevertheless, other authors have shown that the population of these bacteria should be elevated [18,89,101,112]. Strati et al. demonstrated a decline in the presence of *Parabacteroides* in a population with ASD; nevertheless, numerous scientific studies have indicated that the abundance of this bacterium, particularly *P. distasonis*, should be elevated in comparison to healthy populations [18,52,89,101,113]. The scientific research on the genus *Prevotella*, including the species *P. copri*, yielded equivocal results [102,103,107,110,112,114,115]. The potential for an increased prevalence of the family *Prevotellaceae* in the population without ASD has been suggested. However, Li et al. have demonstrated a possibility for a negative correlation between the occurrence of ASD and the family *Prevotellaceae* [52,89,117]. The populations of *Roseburia* and *Turicibacter* have been described as elevated in individuals with ASD by Srikantha et al. and Dargenio et al.; nevertheless, other authors have demonstrated that the population of these bacteria should be decreased [52,89,103,111]. In addition, Li et al. postulate a potential positive correlation between *Turicibacter* and the occurrence of ASD [117]. Furthermore, the results of various analyses indicate that the quantity of *Oscillospira* may be elevated or diminished in the guts of patients diagnosed with ASD [52,101,111,112]. Ristori et al. revealed that individuals diagnosed with ASD exhibited a reduced population of bacteria from the *Ruminococcus* genus; however, numerous studies have demonstrated that the population of these bacteria could be augmented [101,107,109,110]. Furthermore, the research conducted by Coretti et al. and Ristori et al. suggests that, in patients diagnosed with ASD, there is not only an increase in the overall prevalence of *Ruminococcus* bacteria but also a potential elevation in the abundance of *R. torques* species [101,107]. In addition, Li et al. presented the probability of positive associations between the occurrence of ASD and the genus *Ruminococcus* [117]. Further research is needed to clarify the relationship between the bacteria listed in Table 3 and ASD. Authors have proposed that studies should be conducted on specific bacterial species.

## 4. Microbiota-Targeted Therapies in Autism Spectrum Disorders

The complex interaction between the gut microbiota and the central nervous system has garnered significant attention in efforts to understand the pathophysiology of ASD. Feeding abnormalities and food selectivity are also major concerns in ASD, manifesting from infancy as disorganized and vigorous sucking during breastfeeding to avoidant/restrictive food intake disorder (ARFID), which is characterized by severe limitations in both the quantity and variety of consumed food. These issues stem from sensory processing difficulties, behavioral inflexibility, and anxiety, leading to a restricted diet, persistent nutritional deficiencies, and social challenges, with sensory sensitivities playing a pivotal role. ARFID significantly impacts the gut microbiota by disrupting microbial diversity and composition, leading to an increased presence of potentially harmful bacteria, such as *Enterobacterales* and *Bacteroidaceae* (including *Bacteroides vulgatus*), alongside a notable decline in beneficial *Bifidobacterium* species. This dysbiosis may further exacerbate disruptions in the gut–brain axis, reinforcing the bidirectional relationship between gastrointestinal health and neurodevelopmental outcomes. These findings underscore the potential of microbiota-targeted therapies as a promising approach to modulating the gut ecosystem and, in turn, positively influencing neurodevelopmental and behavioral outcomes in individuals with ASD. Moreover, this highlights a critical opportunity for dietary interventions and other potential treatments to restore microbial balance and improve overall well-being in individuals with ASD [87,118].

### 4.1. Dietary Interventions

Diet plays a critical role in shaping the gut microbiome, providing a non-invasive and accessible strategy for managing symptoms associated with ASD. However, the widespread issue of food selectivity among children with ASD presents a significant barrier to implementing effective dietary interventions. Many children with ASD display strong preferences for specific foods while rejecting others, often resulting in unbalanced diets that negatively affect gut microbial diversity, overall health, and nutrient intake. These dietary imbalances frequently lead to deficiencies in essential nutrients such as fiber, iron, calcium, vitamin D, and essential fatty acids, further influencing gut and brain health [118].

The table below (Table 4) presents a concise summary of therapeutic diets that have demonstrated potential benefits in managing autism-related symptoms. Each diet offers unique mechanisms and poses distinct challenges, emphasizing the importance of personalized dietary approaches.

### 4.2. Probiotic Interventions

Probiotics, defined as live microorganisms that confer health benefits when consumed in adequate amounts, have attracted considerable attention for their potential to modulate symptoms associated with ASD. These benefits are attributed to several key mechanisms. First, probiotics produce SCFAs, such as butyrate, which help reduce gut inflammation and strengthen the intestinal barrier, preventing the entry of harmful substances into the bloodstream and potentially mitigating neuroinflammation. Second, probiotics can influence neurotransmitter production, which is essential for social behavior, mood regulation, and cognitive function. By doing so, they may enhance social interactions and reduce anxiety. Third, probiotics contribute to restoring a healthy gut microbiota, addressing dysbiosis that is often associated with both gastrointestinal and behavioral symptoms in ASD. Additionally, probiotics can modulate immune responses, reducing both gut and systemic inflammation, which may alleviate neuroinflammation and improve cognitive functioning. Finally, they can enhance gastrointestinal health by alleviating common symptoms such as constipation and bloating, which are frequently observed in individuals with ASD [126].

A study by Meguid et al. examined the effects of probiotics as an adjuvant therapy in 40 children with ASD, aged 2–5 years. Participants were administered a whey-based nutritional supplement fortified with *Bifidobacterium* spp. and *Lactobacillus* spp. daily for three months. The study reported significant increases in stool colony counts of *Bifidobacterium* spp. (*p* = 0.000) and *Lactobacillus* spp. (*p* = 0.015). Clinically, 80% of children experienced reduced anxiety and improved sleep, alongside better scores on the Childhood Autism Rating Scale (CARS) and the Pediatric Gastrointestinal Symptoms Questionnaire [127].

A randomized, double-blind, placebo-controlled pilot trial evaluated the combined effects of *Lactobacillus plantarum* PS128 (6 × 10^10^ CFUs) and oxytocin in 35 individuals with ASD, aged 3–20 years, over 28 weeks. Participants were administered either probiotics or a placebo for 16 weeks, followed by oxytocin for all groups during weeks 16 to 28. The combination therapy demonstrated significant improvements in Social Responsiveness Scale (SRS), Aberrant Behavior Checklist (ABC), and Clinical Global Impression (CGI) scores compared to the placebo (*p* < 0.05). Moreover, enhanced gut microbiome hubs, particularly the *Eubacterium hallii* group, were positively correlated with improved social cognition [128].

In another randomized, six-month controlled trial, researchers investigated brain activity changes in 46 children with ASD receiving probiotics. Electroencephalography (EEG) analyses revealed decreased power in frontopolar regions (beta and gamma bands), increased coherence, and shifts in frontal asymmetry, indicating brain activity patterns closer to those observed in neurotypical individuals. These EEG changes were significantly correlated with both clinical and biochemical improvements, supporting the potential of probiotics to influence brain activity in ASD. These findings highlight the need for further investigation into the underlying mechanisms [129].

Despite these promising findings, the evidence supporting probiotics as a treatment for ASD remains preliminary. While initial trials suggest potential benefits such as improved gastrointestinal symptoms (e.g., constipation, diarrhea, and bloating), reduced anxiety, and enhanced social behavior, inconsistencies in results highlight the need for further research. Specifically, studies are needed to identify the most effective probiotic strains, optimal dosages, and long-term safety profiles. Larger, well-controlled clinical trials are essential, as are investigations into personalized probiotic treatments tailored to individual microbiota profiles.

### 4.3. Exploring Fecal Microbiota Transplantation as a Novel Therapy for Autism

Fecal microbiota transplantation (FMT) involves transferring the gut microbiota from a healthy donor to a recipient to restore microbial diversity and balance within the gut. Interestingly, research indicates that GI problems are associated with the severity of ASDs [14]. Research suggests that those who may benefit the most include individuals with ASD experiencing chronic GI issues such as persistent constipation, diarrhea, bloating, or irritable bowel syndrome (IBS)-like symptoms [130].

The relationship between maternal and early-life antibiotic exposure and autism risk is still unclear, but patients with a history of antibiotic overuse, which can drastically change the composition of their gut microbiota, may also be candidates for FMT [131,132,133]. Finaly, FMT is a promising alternative for those with ASD who have not responded well to conventional therapy.

A small open-label trial conducted by Kang et al. evaluated the effects of Microbiota Transfer Therapy (MTT) on gut microbiota composition, GI symptoms, and ASD-related behaviors in 18 children with ASD. The therapy protocol included a two-week antibiotic regimen, bowel cleansing, and FMT with an initial high dose followed by daily maintenance doses for 7–8 weeks. Notably, GI symptoms improved by approximately 80%, with significant reductions in constipation, diarrhea, indigestion, and abdominal pain. These benefits were sustained eight weeks after treatment. Behavioral symptoms also improved significantly and remained stable post-treatment. Sequencing analyses revealed the partial engraftment of donor microbiota, increased bacterial diversity, and higher levels of beneficial taxa such as *Bifidobacterium*, *Prevotella*, and *Desulfovibrio*, which persisted following the intervention [134].

In a two-year follow-up study, Kang et al. further assessed the long-term effects of MTT in the same group of 18 children with ASD. The majority of GI symptom improvements were maintained, while autism-related behaviors continued to show positive progress after treatment. Significant gut microbiota changes observed at the end of treatment, including increased bacterial diversity and higher levels of *Bifidobacteria* and *Prevotella*, were still evident at follow-up. These findings highlight the long-term safety and efficacy of MTT as a potential therapeutic option for children with ASD experiencing GI issues [135].

Although FMT shows potential for ASD treatment, several limitations exist. The lack of large-scale, randomized controlled trials makes it challenging to generalize outcomes. Despite some studies having shown benefits regarding behavioral and gastrointestinal issues in the short term, the long-term implications are yet uncertain. Furthermore, individual responses may vary due to variations in gut microbiota, genetics, and environment. Safety considerations should also be considered, such as infection risks and unintended microbiota changes. Further research is essential to validate the efficacy, safety, and long-term effects of FMT in ASD, as well as to better understand its mechanisms, individual variability, and other challenges [136,137].

In the future, it might be feasible to create a tailored FMT for ASD with just advantageous bacterial species. A targeted FMT for ASD in the future would likely include specific beneficial bacterial strains known for their role in restoring gut health, modulating the immune system, and influencing the gut–brain axis. In individuals with ASD, there is often an imbalance in the immune system, with heightened levels of certain pro-inflammatory cytokines or lower concentrations of anti-inflammatory cytokines. Upregulated inflammatory cytokines have been closely associated with ASD behavioral symptoms [138].

Bacteria that could be used in the future may include beneficial species such as Bifidobacterium species, Lactobacillus species, Akkermansia muciniphila, and Prevotella species. These beneficial bacterial strains can help achieve the goals of restoring gut barrier integrity, enhancing SCFA production, modulating immune and inflammatory responses, regulating neurotransmitter production, and improving gut microbial diversity, all of which are crucial for supporting gut health and alleviating ASD-related symptoms [114,139,140,141,142,143,144].

## 5. Challenges and Future Research Directions

Initial research on the influence of the microbiota on ASD suggest that interventions targeting the gut microbiome may provide a promising approach for alleviating symptoms and addressing coexisting gastrointestinal issues. This potential arises from the microbiota’s ability to modulate inflammation [89]. In recent years, increasing attention has been given to the significant role of dysbiosis in the etiopathogenesis of disorders linked to the central nervous system. The inflammatory changes observed in conditions such as schizophrenia, major depressive disorder, and bipolar disorder highlight microbiota disturbances and underscore the gastrointestinal system’s fundamental role in the development of neuropsychiatric disorders. The involvement of gut microorganisms in these conditions has been extensively studied through numerous animal models. These studies include comparisons of gut microbiota composition between affected individuals and control groups, assessments of behavioral changes following the use of microbiota modulators in affected subjects, and investigations into the impact of virulence factors on control groups [110].

Emerging research increasingly emphasizes clinical interventions aimed at restoring gut microbiota balance through antibiotics, prebiotics, probiotics, and FMT. However, the diversity and heterogeneity of existing clinical studies pose challenges regarding establishing a robust evidence base for developing targeted therapies. This underscores the need for standardized methods to assess gut microbiota composition and inflammation levels, which are essential for reliably evaluating the effects of medical interventions [89]. Future studies should prioritize investigating the long-term effects of microbiota-focused therapies across different life stages in individuals with ASD. Additionally, identifying specific metabolites produced by gut microorganisms that influence neurological development and function is critical. Another key objective is to develop personalized therapeutic strategies tailored to each individual’s unique microbiota composition, genetic profile, dietary habits, and coexisting health conditions. Furthermore, optimizing probiotic treatments remains a challenge, particularly in determining the most effective bacterial species, subspecies, and dosages. A comprehensive, multidisciplinary approach should emphasize psychoeducational and therapeutic interventions, including behavioral strategies that actively involve both patients and their caregivers. Parents who acknowledge and voice concerns about their child’s weight issues are often more driven to make positive changes in the family’s diet and actively support healthier eating and physical activity habits for their child [87].

Addressing these challenges will be essential for advancing this field in the coming years, with a strong emphasis on personalized therapeutic interventions incorporating specific microbiome selections and other proposed treatments [145].

## 6. Conclusions

The connection between gut microbiota and ASD represents a promising avenue for understanding and addressing the complexities of this condition. Research indicates that dysbiosis in the gut microbiota not only contributes to gastrointestinal symptoms but also plays a significant role in shaping behavioral and neuropsychological outcomes in individuals with ASD [8,52,89]. Dietary interventions show potential in improving gut health and reducing inflammation, which may alleviate ASD symptoms [119,124,125]. Similarly, probiotic therapies and FMT have demonstrated efficacy in enhancing microbial diversity, reducing digestive issues, and improving behavioral functioning [134,135,145]. However, the variability in clinical responses highlights the need for personalized approaches that consider individual microbiota composition, genetics, diet, and coexisting conditions [89,145]. Future research should focus on standardizing microbiota assessment methods, identifying the most effective microbial strains, and evaluating the long-term safety and efficacy of microbiota-targeted therapies [89,145]. By integrating these strategies with a deeper understanding of the gut–brain axis, innovative treatment approaches can be developed to significantly enhance the quality of life for individuals with ASD and their families [52,89,145].

## Figures and Tables

**Table 1:** Analysis of bacterial populations reveals a higher quantity in patients diagnosed with ASD when compared to healthy individuals. The table provides a comprehensive overview of the distribution of successive taxonomic levels, encompassing cluster, class, order, family, and genus.

Phylum	Class	Order	Family	Genus	Gram Classification	Presence in ASD
* Actinomycetota *	*Coriobacteriia*	*Coriobacteriales*	*Coriobacteriaceae*	*Collinsella*	Positive	Higher [18]
* Actinomycetota *	*Coriobacteriia*	*Eggerthellales*	*Eggerthellaceae*	*Eggerthella*	Positive	Higher [104]
* Bacillota *	*Clostridia*	*Clostridiales*	*Clostridiaceae*	*Caloramator*	Positive	Higher [8,50,96,97,98]
* Bacillota *	*Clostridia*	*Clostridiales*	*Clostridiaceae*	*Sarcina*	Positive	Higher [8,50,96,97,98]
* Bacillota *	*Clostridia*	*Eubacteriales*	*Lachnospiraceae*	-	Positive	Higher [105]
* Bacillota *	*Clostridia*	*Eubacteriales*	*Lachnospiraceae*	*Dorea*	Positive	Higher [18,52]
* Bacillota *	*Clostridia*	*Eubacteriales*	*Oscillospiraceae*	*Anaerofilum*	Positive	Higher [52]
* Bacillota *	*Clostridia*	*Eubacteriales*	*Oscillospiraceae*	*Faecalibacterium*	Positive	Higher [52,89,101,107,109,113]
* Bacillota *	*Clostridia*	*Eubacteriales*	*Peptostreptococcaceae*	*Peptostreptococcus*	Positive	Higher [112]
* Bacteroidota *	*Bacteroidia*	*Bacteroidales*	*Bacteroidaceae*	*Bacteroides*	Negative	Higher [101,103,108,109,113]
* Bacteroidota *	*Bacteroidia*	*Bacteroidales*	*Barnesiellaceae*	*Barnesiella*	Negative	Higher [52]
* Bacteroidota *	*Bacteroidia*	*Bacteroidales*	*Odoribacteraceae*	*Odirobacter*	Negative	Higher [52]
* Bacteroidota *	*Bacteroidia*	*Bacteroidales*	*Porphyromonadaceae*	*Porphyromonas*	Negative	Higher [52]
* Bacteroidota *	*Bacteroidia*	*Bacteroidales*	*Rikenellaceae*	*Alistipes*	Negative	Higher [50,89,96,97,98]
* Firmicutes *	*Bacilli*	*Lactobacillales*	*Lactobacillaceae*	*Lactobacillus*	Positive	Higher [8,18,50,96,97,98,102,103,106,109,110,111]
* Firmicutes *	*Clostridia*	*Clostridiales*	*Ruminococcaceae*	-	Positive	Higher [8,101]
* Firmicutes *	*Clostridia*	*Clostridiales*	*Clostridiaceae*	*Clostridium*	Positive	Higher [8,50,52,96,97,98,99,100,101,102,103,104,105,106,107,108,109]
* Proteobacteria *	*Deltaproteobacteria*	*Desulfovibrionales*	*Desulfovibrionaceae*	*Desulfovibrio*	Negative	Higher [50,96,97,98,103,106,107,109]
* Pseudomonadota *	*Gammaproteobacteria*	*Enterobacteriales*	*Enterobacteriaceae*	-	Negative	Higher [8,50,52,96,97,98,101,111]
* Pseudomonadota *	*Gammaproteobacteria*	*Pasteurellales*	*Pasteurellaceae*	-	Negative	Higher [8,101]
* Pseudomonadota *	*Gammaproteobacteria*	*Pasteurellales*	*Pasteurellaceae*	*Haemophilus*	Negative	Higher [102]
* Pseudomonadota *	*Gammaproteobacteria*	*Enterobacteriales*	*Enterobacteriaceae*	*Klebsiella*	Negative	Higher [104]
* Pseudomonadota *	*Betaproteobacteria*	*Burkholderiales*	*Sutterellaceae*	*Parasutterella*	Negative	Higher [52]
* Pseudomonadota *	*Gammaproteobacteria*	*Aeromonadales*	*Aeromonadaceae*	*Aeromonas*	Negative	Higher [52]
* Pseudomonadota *	*Gammaproteobacteria*	*Pseudomonadales*	*Pseudomonadaceae*	*Pseudomonas*	Negative	Higher [52]
* Pseudomonadota *	*Betaproteobacteria*	*Burkholderiales*	*Alcaligenaceae*	-	Negative	Higher [52,110]
* Pseudomonadota *	*Betaproteobacteria*	*Burkholderiales*	*Burkholderiaceae*	*Burkholderia*	Negative	Higher [112]
* Pseudomonadota *	*Betaproteobacteria*	*Burkholderiales*	*Burkholderiaceae*	*Ralstonia*	Negative	Higher [112]
* Pseudomonadota *	*Betaproteobacteria*	*Burkholderiales*	*Sutterellaceae*	*Sutterella*	Negative	Higher [89,107,109,110]
* Pseudomonadota *	*Alphaproteobacteria*	*Hyphomicrobiales*	*Phyllobacteriaceae*	*Phyllobacterium*	Negative	Higher [111]
* Verrucomicrobiota *	*Verrucomicrobiae*	*Verrucomicrobiales*	*Akkermansiaceae*	*Akkermansia*	Negative	Higher [52,89,107]

The nomenclature of bacterial taxa is indicated by italics.

**Table 2:** Analysis of bacterial populations reveals a lower quantity in patients diagnosed with ASD when compared to healthy individuals. The table provides a comprehensive overview of the distribution of successive taxonomic levels, encompassing cluster, class, order, family, and genus.

Phylum	Class	Order	Family	* Genus *	Gram Classification	Presence in ASD
* Actinobacteria *	*Coriobacteriia*	*Coriobacteriale*	*Coriobacteriaceae*	-	Positive	Lower [8,101]
* Actinomycetota *	*Actinomycetia*	*Actinomycetales*	*Actinomycetaceae*	-	Positive	Lower [8,101]
* Actinomycetota *	*Actinomycetia*	*Bifidobacteriales*	*Bifidobacteriaceae*	-	Positive	Lower [8,101]
* Actinomycetota *	*Actinomycetia*	*Bifidobacteriales*	*Bifidobacteriaceae*	*Bifidobacterium*	Positive	Lower [52,89,101,102,103,106,107,113]
* Bacillota *	*Bacilli*	*Caryophanales*	*Staphylococcaceae*	*Staphylococcus*	Positive	Lower [107]
* Bacillota *	*Clostridia*	*Eubacteriales*	*Lachnospiraceae*	*Coprococcus*	Positive	Lower [8,52,107,111,114,115]
* Bacillota *	*Negativicutes*	*Vellionellales*	*Veillonellaceae*	*Veillonella*	Negative	Lower [8,18,52,114,115]
* Bacillota *	*Bacilli*	*Lactobacillales*	*Streptococcaceae*	*Streptococcus*	Positive	Lower [8,52,101,107,112]
* Bacillota *	*Clostridia*	*Eubacteriales*	*Lachnospiraceae*	*Blautia*	Positive	Lower [103]
* Bacillota *	*Negativicutes*	*Veillonellales*	*Veillonellaceae*	*Dialister*	Negative	Lower [18,103]
* Bacillota *	*Clostridia*	*Eubacteriales*	*Oscillospiraceae*	*Subdoligranulum*	Positive	Lower [52]
* Bacillota *	*Bacilli*	*Lactobacillales*	*Streptococcaceae*	*Lactococcus*	Positive	Lower [52,107]
* Bacillota *	*Clostridia*	*Eubacteriales*	*Oscillospiraceae*	*Flavonifractor*	Positive	Lower [111]
* Bacillota *	*Clostridia*	*Clostridiales*	*Eubacteriaceae*	*Eubacterium*	Positive	Lower [109]
* Firmicutes *	*Clostridia*	*Clostridiales*	*Ruminococcaceae*	*Sporobacter*	Positive	Lower [52]
* Firmicutes *	*Bacilli*	*Lactobacillales*	*Enterococcaceae*	*Enterococcus*	Positive	Lower [52,107,109]
* Fusobacteriota *	*Fusobacteriia*	*Fusobacteriales*	*Fusobacteriaceae*	*Fusobacterium*	Negative	Lower [52]
* Pseudomonadota *	*Alphaproteobacteria*	*Hyphomicrobiales*	*Devosiaceae*	*Devosia*	Negative	Lower [112]
* Pseudomonadota *	*Betaproteobacteria*	*Neisseriales*	*Neisseriaceae*	*Neisseria*	Negative	Lower [112]
* Pseudomonadota *	*Gammaproteobacteria*	*Enterobacterales*	*Enterobacteriaceae*	*Escherichia*	Negative	Lower [52,112]
* Thermodesulfobacteriota *	*Desulfovibrionia*	*Desulfovibrionales*	*Desulfovibrionaceae*	*Bilophila*	Negative	Lower [18]

The nomenclature of bacterial taxa is indicated by italics.

**Table 3:** The following table contains a list of bacterial classifications, including phylum, class, order, family, genus, and Gram classifications, for which researchers have obtained divergent gut microbiota results in patients with ASD compared to healthy populations.

Phylum	Class	Order	Family	Genus	Gram Classification	Higher Presence in ASD	Lower Presence in ASD
* Actinomycetota *	*Actinomycetia*	*Actinomycetales*	*Actinomycetaceae*	*Actinomyces*	Positive	[89,112]	[101]
* Actinomycetota *	*Actinomycetia*	*Mycobacteriales*	*Corynebacteriaceae*	*Corynebacterium*	Positive	[18]	[101]
* Bacillota *	*Clostridia*	*Eubacteriales*	*Oscillospiraceae*	*Oscillospira*	Positive	[101,111,112]	[52]
* Bacillota *	*Clostridia*	*Eubacteriales*	*Lachnospiraceae*	*Roseburia*	Positive	[52,89]	[111]
* Bacillota *	*Erysipelotrichia*	*Erysipelotrichales*	*Turicibacteraceae*	*Turicibacter*	Positive	[52,89]	[103]
* Bacteroidetes *	*Bacteroidia*	*Bacteroidales*	*Prevotellaceae*	*Prevotella*	Negative	[110,114,115]	[102,103,107,112]
* Bacteroidetes *	*Bacteroidia*	*Bacteroidales*	*Porphyromonadaceae*	*Parabacteroides*	Negative	[52,89,113]	[18]
* Firmicutes *	*Clostridia*	*Clostridiales*	*Ruminococcaceae*	*Ruminococcus*	Positive	[101,109,110]	[107]

The nomenclature of bacterial taxa is indicated by italics.

**Table 4:** The overview of therapeutic diets for managing autism-related symptoms.

Diet Type	Key Features	Mechanism of Action	Reported Benefits	Key References
Gluten-Free Diet	Elimination of gluten, a protein found in wheat, barley, and rye	Prevention of gluten-derived opioid-like peptides; reduction in intestinal permeability and systemic inflammation	Reduction in GI symptoms and hyperactivity, and improvements in social behavior and focus	[119,120,121]
Casein-Free Diet	Elimination of casein, a protein found in dairy products	Reduction in casein-derived opioid-like peptides; minimization of GI inflammation and intestinal permeability	Improvement in GI disturbances and potential behavioral benefits	[120,121]
Ketogenic Diet	High-fat, low-carbohydrate intake	Ketone production as alternative brain energy source; reduction in neuroinflammation and oxidative stress	Seizure reduction (in co-occurring epilepsy), improved attention, and behavioral enhancements	[122,123]
High-Antioxidant Diet	Emphasis on antioxidant-rich foods such as fruits, vegetables, and nuts	Neutralization of reactive oxygen species; reduction in oxidative stress and systemic/neural inflammation	Behavioral enhancement, oxidative damage reduction, and neuroprotection	[118,124]
Prebiotic-Rich Diet	Incorporation of fermented foods like yogurt, kefir, and sauerkraut	Restoration of microbial balance; enhancement of gut integrity; production of metabolites influencing brain function	Reduction in GI symptoms, anxiety, and improved social interactions	[118,125]

## Data Availability

The data used in this article is sourced from materials mentioned in the References section.
